# Preoperative red cell distribution width is associated with postoperative lymphovascular invasion in prostate cancer patients treated with radical prostatectomy: A retrospective study

**DOI:** 10.3389/fendo.2022.1020655

**Published:** 2022-10-13

**Authors:** Fangming Wang, Jing Liang, Feiya Yang, Fei Liu, Sujun Han, Nianzeng Xing

**Affiliations:** ^1^ Department of Urology, National Cancer Center/National Clinical Research Center for Cancer/Cancer Hospital, Chinese Academy of Medical Sciences and Peking Union Medical College, Beijing, China; ^2^ State Key Laboratory of Molecular Oncology, National Cancer Center/National Clinical Research Center for Cancer/Cancer Hospital, Chinese Academy of Medical Sciences and Peking Union Medical College, Beijing, China; ^3^ Department of Pathology, National Cancer Center/National Clinical Research Center for Cancer/Cancer Hospital, Chinese Academy of Medical Sciences and Peking Union Medical College, Beijing, China; ^4^ Department of Urology, Shanxi Province Cancer Hospital/Shanxi Hospital Affiliated to Cancer Hospital, Chinese Academy of Medical Sciences/Cancer Hospital Affiliated to Shanxi Medical University, Taiyuan, China

**Keywords:** red blood cell distribution width, lymphovascular invasion, prostate cancer, radical prostatectomy, hematological parameters

## Abstract

**Purpose:**

To investigate the relationship between baseline clinicopathological and laboratory variables especially hematological parameters and lymphovascular invasion (LVI) in patients who underwent radical prostatectomy (RP).

**Methods:**

We retrospectively evaluated 348 prostate cancer (PCa) patients who underwent RP in our center between May 2018 and June 2021. We divided them into non-LVI and LVI groups based on LVI status, and compared clinicopathological characteristics between non-LVI and LVI groups. Clinicopathological parameters including age, body mass index (BMI), history of hypertension and diabetes mellitus, neoadjuvant hormonal therapy (NHT), pathological stage T (pT) and lymph node status (pN), ISUP (international society of urological pathology) grade, positive surgical margin (PSM) rate, and hematological parameters containing prostate-specific antigen (PSA), whole blood parameters and inflammatory indexes were collected. The association between the clinicopathological parameters and the presence of LVI was identified by multivariate logistic regression analysis.

**Results:**

The pathological results of the RP specimen consisted of 53 (15.2%) patients with LVI and 295 (84.8%) cases without LVI. The level of PSA, percentages of advanced pT and grade, pN1, and PSM were significantly higher in the LVI group when compared with the non-LVI counterpart (p<0.001, p<0.001, p<0.001, p<0.001, p=0.007, respectively). Among the whole blood parameters, only red cell distribution width (RDW) was significantly different (41.2 ± 2.5 vs. 42.1 ± 3.1, p=0.035). Multivariate regression analysis demonstrated that RDW and NHT were negatively correlated with the presence of LVI (OR = 0.870, p=0.024; OR = 0.410, p=0.025), while PSA, ISUP, and pT were positively correlated with the presence of LVI (OR=1.013, p=0.005; OR =1.589, p=0.001; OR=1.655, p=0.008) after adjusting for confounding factors.

**Conclusions:**

RDW rather than other whole blood parameters was independently and negatively associated with the presence of LVI in PCa patients, suggesting that RDW might play an essential role in PCa invasion.

## Introduction

Worldwide, prostate cancer (PCa) represents the second most common solid tumor in men. There were over 1414000 estimated new cases of PCa worldwide in the year 2020, with an age-standardized rate (ASR) incidence of 31 per 100000. Besides, there were over 375000 estimated deaths worldwide, and the overall mortality ASR was 7.7 per 100000 (1). Radical prostatectomy (RP) is the most common treatment options for localized PCa, and increasingly used as an important step for the treatment of advanced local, and even early metastatic cases when indicated (2). The pathology report of RP specimens including stage, positive surgical margin (PSM), grade, perineural invasion (PNI), lymphovascular invasion (LVI), etc. is critical in accurately determining the prediction of patient outcome (3). More and more digital pathological information assisted by artificial intelligence (AI) and radiomics was explored and added to the current models for improving the risk stratification and the prediction of survival and treatment response (4, 5). Recently, LVI and PNI, the two important pathologic parameters which can be assisted by AI, attracted more and more attention in multiple malignancies (6). We have previously explored the relationship between clinicopathological parameters and PNI in patients who underwent RP (7). Next, we focused on factors influencing LVI on RP specimen.

LVI is a well-recognized histopathologic parameter that is associated with unfavorable prognosis in malignancies (8, 9). LVI includes lymphatic invasion, vascular invasion, or both, which is considered as a critical step in priming metastasis (www.cap.org/protocols-and-guidelines/cancer-reporting-tools/cancer-protocoltemplates). For PCa, LVI is reported in up to 21.5% of RP specimens (www.cap.org/protocols-and-guidelines/cancer-reporting-tools/cancerprotocoltemplates), and the presence of LVI at time of RP is recognized as an adverse pathological feature (10–12). Furthermore, several studies have observed that LVI could mirror lymph node invasion status (13), and was associated with unfavorable biochemical recurrence (BCR) rates and overall survival after RP (11, 12, 14, 15). The underlying mechanism of LVI remains unclear although some recent studies tried to reveal it from genetic level (16–18). It involves not only the acquisition of more invasive and migration abilities of the malignant epithelial cells but also the interaction with the surrounding tumor microenvironment (TME) along with endothelial cells lining the lymphovascular spaces (19).

It is well-known that hemocytes, including red blood cell (RBC), white blood cell (WBC), and platelet (PLT), provide basic elements to conceive the immune and inflammatory TME, which consists of distinct immune cell populations highly associated with the antitumor immunological state in tumor islets (20). Moreover, some indicators in or derived from hematological parameters in routine blood test, such as red cell distribution width (RDW) (21), neutrophil-to-lymphocyte ratio (N/L), platelet-to-lymphocyte ratio (P/L), systemic immune-inflammation index (SII), lymphocyte-to-monocyte ratio (L/M), and systemic inflammation response index (SIRI), have attracted more and more attention in recent years due to their potential role as diagnostic or prognostic markers (22–26). However, the associated clinicopathological characteristics of LVI in PCa remain largely unknown. Till now, no data are available on the correlation of the clinicopathological parameters with LVI.

Therefore, our objective was to systematically and comprehensively evaluate the association of the clinicopathological parameters especially the whole blood parameters with the presence of LVI of RP specimen in the Chinese patients with PCa.

## Patients and methods

### Study subjects

The current study included patiens who underwent laparoscopic RP and pelvic lymph node dissection in our center between May 2018 and June 2021. The exclusion criteria of the study were the presence of hematologic diseases, anemia, acute or chronic infection, severe hepatic and/or renal insufficiency, hypersplenism, hyperthyroidism, cardiovascular disease, other malignant tumors or a history of other malignancies, preoperative radiotherapy or chemotherapy. Finally, a total of 348 cases were included.

### Collection of patients’ clinicopathological data

All demographic and clinicopathological data were collected from the Cancer Hospital, Chinese Academy of Medical Sciences and Peking Union Medical College information system.

The data included age, body mass index (BMI), hypertension, diabetes mellitus, prostate-specific antigen (PSA), neoadjuvant hormonal therapy (NHT), operative time, blood loss, transfusion, preoperative whole blood parameters, and postoperative pathological results including pathological stage T (pT) and lymph node status (pN), ISUP (international society of urological pathology) grade, PSM. The assessments of cancer pT, pN, and grade were performed as we previously reported (7). The pathology of the presence of LVI, PNI, and PSM was reviewed by 2 senior pathologists through comprehensive analysis of H&E staining results. Diagnostic criterion for LVI was defined as the presence of tumor cells within an endothelial-lined space that is usually devoid of a muscular wall (www.cap.org/protocols-and-guidelines/cancer-reporting-tools/cancer-protocoltemplates).

### Hematological parameters measurement

Routine preoperative whole blood tests were performed, and included WBC, neutrophils (%), neutrophil counts, lymphcytes (%), lymphocyte counts, monocytes (%), monocyte counts, eosinophils (%), eosinophil counts, basophils (%), basophil counts, RBC, hemoglobin (Hb), hematocrit (Hct), mean corpuscular volume (MCV), mean corpuscular hemoglobin (MCH), mean corpuscular hemoglobin concentration (MCHC), RDW-standard deviation (RDW-SD), PLT, mean PLT volume (MPV), PLT-large cell ratio (P-LCR), PLT distribution width (PDW), and plateletcrit. Based on peripheral blood cell counts, systemic inflammation markers were calculated as follows: N/L=neutrophil counts/lymphocyte counts, P/L=platelet counts/lymphocyte counts, SII=(neutrophil counts *platelet counts)/lymphocyte counts, L/M=lymphocyte counts/monocyte counts, SIRI=neutrophil counts×monocyte counts/lymphocyte counts.

### Statistical analysis

Data were expressed as means ± SD or median with interquartile range for continuous variables and number (percentage) for categorical variables. The differences between continuous variables were analyzed by unpaired t-tests or Mann-Whitney U tests as appropriate. Categorical variables were analyzed by χ2-test.

All PCa subjects were divided into two groups according to LVI status: non-LVI and LVI groups, and clinicopathological variables especially hematological parameters were compared between the two groups. The RDW was stratified into quartiles as following: the first quartile group (Q1, <39.8 fl (25th percentile), *n*= 93); the second quartile group (Q2, 39.8-41.7 fl (25-50th percentile), *n* =82); the third quartile group (Q3, 41.7-43.8 fl (50-75th percentile); *n*= 92); the fourth quartile group (Q4, >43.8 fl (75-100th percentile); *n*= 81). Significant clinicopathological risk factors for LVI were analyzed by multivariate logistic regression analysis in 4 models step by step. Model 1 was unadjusted; Model 2 corrected for covariates including age, BMI, and comorbidities.; Model 3 additionally corrected for PCa specific risk factors including PSA, ISUP grade, and stage based on Model 2; Model 4 further corrected for NHT, the intervention before RP surgery, based on Model 3. All tests were two-sided and a p-value <0.05 was considered significant. The statistical analyses were performed with SPSS version 22.0 software (Chicago, IL, USA).

## Results

### Comparison of clinicopathological parameters between non-LVI and LVI groups

**Figure 1:**
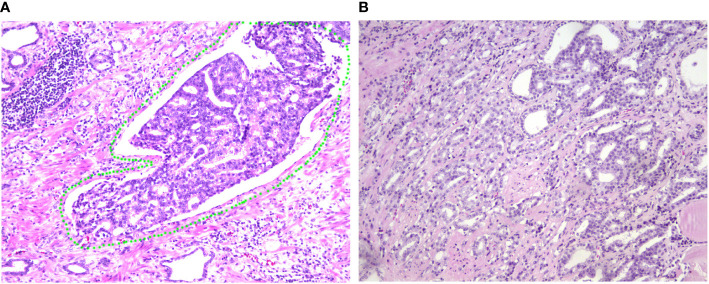
Representative of LVI and non-LVI sections of HE staining from patients with PCa. **(A)** Representative image of LVI (HE 200×). The LVI zone was labelled with a green circle, indicating that the involved vessel is full of PCa cells. **(B)** Representative image of non-LVI (HE 200×) for comparison. LVI, lymphovascular invasion; HE, hematoxylin-eosin; PCa, prostate cancer.

**Figure 2:**
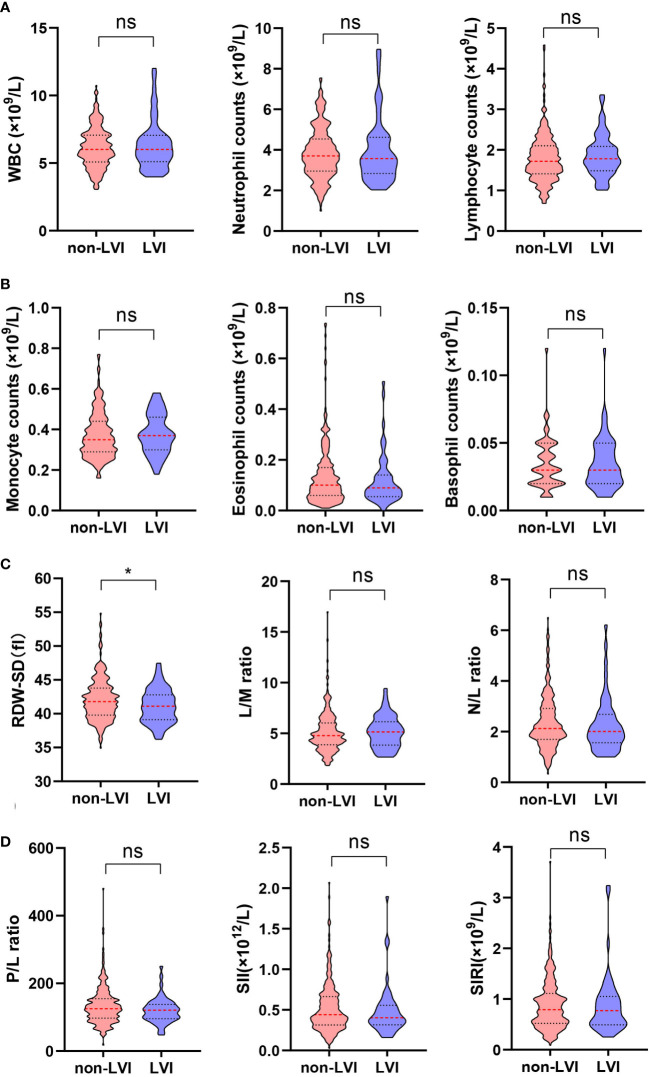
Comparison of RDW-SD and other inflammatory indicators from hematological parameters between non-LVI (n=295) and LVI (n=53) groups. **(A)** (left) WBC, (middle) neutrophil counts, (right) lymphocyte counts; **(B)** (left) monocyte counts, (middle) eosinophil counts, (right) basophil counts; **(C)** (left) RDW-SD, (middle) L/M ratio, (right) N/L ratio; **(D)** (left) P/L ratio, (middle) SII, (right) SIRI. Data are expressed as median with interquartile range and showed in a violin plot (the dotted line), statistical significance was determined by the Mann-Whitney U test. ns, not significant, *, P < 0.05. RDW-SD, red cell distribution width-standard deviation; LVI, lymphovascular invasion; WBC, white blood cell; L/M, lymphocyte-to-monocyte; N/L, neutrophil-to-lymphocyte; P/L, platelet-to-lymphocyte; SII, systemic immune-inflammation index; SIRI, systemic inflammation response index.

**Figure 3:**
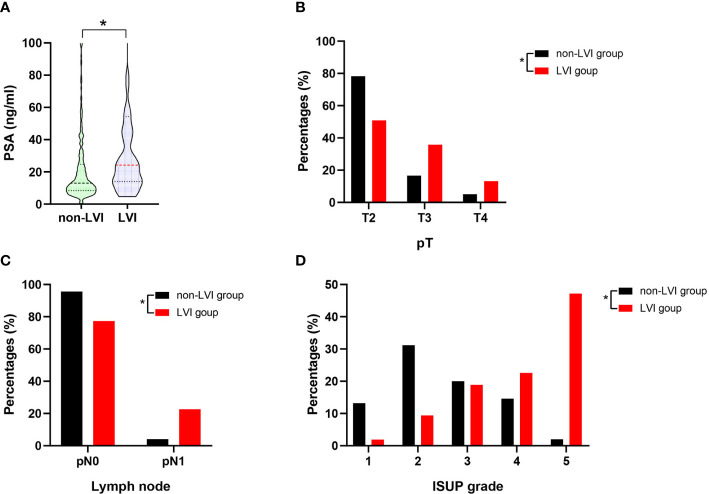
The summary data of PSA level, the percentage distributions of pT, pN1 and IUSP grade in the non-LVI (n=295) and LVI (n=53) groups. **(A)** Comparison of PSA level between non-LVI and LVI groups, statistical significance was determined by Mann-Whitney U test; **(B)** Comparison of T2, T3, and T4 percentages between non-LVI and LVI groups, statistical significance was determined by χ2-test; **(C)** Comparison of pN0 and pN1 percentages between non-LVI and LVI groups, statistical significance was determined by χ2-test; **(D)** Comparison of ISUP grade 1, 2, 3, 4, 5 percentages between non-LVI and LVI groups, statistical significance was determined by χ2-test. Values are expressed as median with interquartile range and showed in a violin plot (the dotted line) or percentage, *, P < 0.05. PSA, prostate-specific antigen; pT, pathological stage T; pN0, pathological lymph node negative; pN1, pathological lymph node positive; ISUP, international society of urological pathology.

The clinicopathological characteristics including whole blood parameters of the non-LVI and LVI groups were shown in **Table 1**. Of the total 348 eligible RP cases, LVI was presented in 53 cases (15.2%), and absent in 295 cases (84.8%). The representative hematoxylin-eosin (HE) staining images of LVI and non-LVI were shown in **Figures 1A, B**. No significant differences in age, BMI, hypertension, diabetes, NHT percentage, metastasis, operative time, blood loss, transfusion, or whole blood parameters except RDW-SD were observed between the non-LVI and LVI groups. Of note, RDW-SD was significantly higher in the non-LVI group than that in the LVI group (42.1 ± 3.1 vs. 41.2 ± 2.5, p=0.035) (**Figure 2**). However, there were no significant difference between the non-LVI and LVI groups regarding inflammatory parameters including WBC, neutrophil counts, lymphocyte counts, monocyte counts, eosinophil counts, basophil counts, N/L, P/L, SII, L/M, and SIRI (**Figure 2**). The level of PSA was significantly higher in the LVI group compared to the non-LVI counterpart [24.2 (14.1-54.3) vs. 13.0 (8.5-24.7), p<0.001] (**Figure 3A**). As shown in **Figures 3B, C**, the percentages of advanced pT (T3, T4) and lymph node involvement were significantly higher in the LVI group when compared with non-LVI counterpart (35.8% vs. 16.6%, 13.2% vs.5.1%, p<0.001; 22.6% vs. 4.1%, p<0.001). Furthermore, the percentages of high IUSP grades (4, 5) were significantly higher in the LVI group when compared with non-LVI counterpart (22.6% vs. 14.6%, 47.2% vs. 2.0%, p<0.001) (**Figure 3D**). In addition, there were also significant differences in the PNI and PSM rates between the two groups (94.3% vs. 69.2%, p<0.001; 43.4% vs. 24.1%, p=0.007).

**Table 1:** Baseline characteristics of the PCa subjects underwent RP according to the LVI stratification.

	Non-LVI (n = 295)	LVI (n = 53)	p value
**Demographic characteristics**			
Age (years)	66.2±6.5	65.9±7.1	0.769
BMI (kg/m^2^)	25.0±3.2	25.9±2.6	0.053
Hypertension (n (%))	135 (45.8)	18 (34.0)	0.133
Diabetes mellitus (n (%))	58 (19.7)	11 (20.8)	0.852
**Clinicopathological parameters**			
PSA (ng/mL)	13.0 (8.5-24.7)	24.2 (14.1-54.3)	**<0.001**
NHT (n (%))	111 (37.6)	23 (43.4)	0.446
Pathological Stage(n (%))			**<0.001**
T2	231 (78.3)	27 (50.9)	
T3	49 (16.6)	19 (35.8)	
T4	45 (5.1)	7 (13.2)	
N	12 (4.1)	12 (22.6)	**<0.001**
M (n (%))	14 (4.7)	5 (9.4)	0.185
ISUP grade (n (%))			**<0.001**
1	39 (13.2)	1 (1.9)	
2	92 (31.2)	5 (9.4)	
3	59 (20.0)	10 (18.9)	
4	43 (14.6)	12 (22.6)	
5	62 (2.0)	25 (47.2)	
PNI (n (%))	204 (69.2)	50 (94.3)	**<0.001**
PSM (n (%))	71 (24.1)	23 (43.4)	**0.007**
Operative time (minutes)	163.3±55.2	176.2±72.0	0.137
Evaluated blood loss (ml)	57.0±86.5	73.0±130.0	0.390
Transfusion (n (%))	5 (1.7)	1 (1.9)	0.921
**Whole blood parameters**			
WBC (×10^9^/L)	6.1±1.5	6.3±1.8	0.373
Neutrophils (%)	61.4±9.4	61.0±8.9	0.734
Neutrophil counts (×10^9^/L)	3.8±1.2	4.0±1.6	0.513
Lymphocytes (%)	29.6±8.7	30.4±7.7	0.546
Lymphocyte counts (×10^9^/L)	1.8±0.6	1.9±0.5	0.344
Monocytes (%)	6.1±1.5	6.1±1.5	0.978
Monocyte counts (×10^9^/L)	0.4±0.1	0.4±0.1	0.703
Eosinophils (%)	2.2±1.9	2.0±1.7	0.342
Eosinophil counts (×10^9^/L)	0.1±0.1	0.1±0.1	0.386
Basophils (%)	0.6±^±^0.3	0.5±0.3	0.370
Basophil counts (×10^9^/L)	0.04±0.02	0.03±0.02	0.523
RBC (×10^12^/L)	4.7±0.5	4.8±0.5	0.459
Hb (g/L)	147.2±13.7	147.8±14.8	0.801
Hct (L/L)	0.43±0.04	0.43±0.04	0.951
MCV (fl)	91.7±5.0	90.6±4.9	0.149
MCH (pg)	31.3±3.7	30.9±2.1	0.362
MCHC (g/L)	338.5±20.3	340.3±12.7	0.538
RDW-SD (fl)	42.1±3.1	41.2±2.5	**0.035**
PLT (×10^9^/L)	213.0 (179.0-248.0)	207.0 (175.0-248.0)	0.712
MPV (fl)	10.0±1.1	9.9±0.8	0.649
P-LCR (%)	24.9±7.6	23.9±6.1	0.396
PDW (fl)	11.3±2.2	11.0±1.6	0.351
PCT (×10^-3^L/L)	2.1 (1.8-2.5)	2.1(1.8-2.5)	0.712
N/L	2.1 (1.7-2.9)	2.0 (1.6-2.7)	0.320
P/L	125.1 (97.2-154.9)	120.6 (95.6-137.7)	0.124
SII (×10^9^/L)	439.7 (313.9-664.4)	404.3 (315.4-557.1)	0.251
L/M	4.8 (3.9-6.0)	5.1 (3.8-6.1)	0.426
SIRI (×10^9^/L)	0.8 (0.5-1.1)	0.8 (0.5-1.0)	0.546

Data are expressed as n (%), mean ± SD, or median (interquartile range). The bold value indicated statistical significance. PCa, prostate cancer; RP, radical prostatectomy; LVI, lymphovascular invasion; BMI, body mass index; PSA, prostate-specific antigen; NHT, neoadjuvant hormonal therapy; M, metastasis; ISUP, International Society of Urological Pathology; PNI, perineural invasion; PSM, positive surgical margin; WBC, white blood cell; RBC, red blood cell; Hb, hemoglobin; Hct, hematocrit; MCV, mean corpuscular volume; MCH, mean corpuscular hemoglobin; MCHC, mean corpuscular hemoglobin concentration; RDW-SD, red blood cell volume distribution width-standard deviation; PLT, platelet; MPV, mean platelet volume; P-LCR, platelet-large cell ratio; PDW, platelet distribution width; PCT, plateletcrit; N/L, neutrophil-to-lymphocyte ratio; P/L, platelet-to-lymphocyte ratio; SII, systemic immune-inflammation index; L/M, lymphocyte-to-monocyte ratio; SIRI, systemic inflammation response index.

### Correlations of RDW and other clinicopathological variables with the presence of LVI

We performed multivariate logistic regression analysis to evaluate the correlations of RDW and other clinical variables with LVI. As shown in **Table 2**, RDW-SD was found to be significantly and negatively correlated with the presence of LVI (OR=0.890, 95% CI: 0.799-0.992, p=0.036) in univariate logistic regression analysis. In the stepwise multivariate regression analysis, we gradually added and adjusted confounding factors from model 1 to model 4, and ultimately revealed that the correlations of RDW, NHT, PSA, ISUP, and pT with LVI were significant after adjustment for confounding factors (OR=0.870, 95% CI: 0.770-0.982, p=0.024; OR=0.410, 95% CI: 0.187-0.896, p=0.025; OR=1.013, 95% CI: 1.004-1.022, p=0.005; OR=1.589, 95% CI: 1.197-2.110, p=0.001; OR=1.655, 95% CI: 1.138-2.406, p=0.008, respectively). It is noteworthy that RDW and NHT were negatively correlated with the presence of LVI, while PSA, ISUP, and pT were positively correlated with the presence of LVI.

**Table 2:** Multivariate analysis to identify the independent correlation between RDW and LVI.

Models	Variables	Multivariate mode		p value
				OR	95% CI	
**1**	**unadjusted**			
	RDW-SD	**0.890**	**0.799-0.992**	**0.036**
**2**	**Model 1+covariates**			
	RDW-SD	**0.890**	**0.797-0.994**	**0.039**
	Age	1.009	0.964-1.057	0.689
	BMI	**1.110**	**1.006-1.224**	**0.037**
	Hypertension	0.540	0.286-1.020	0.058
	DM	1.054	0.500-2.220	0.890
**3**	**Model 2+PSA+ ISUP+ T stage+ M**			
	RDW-SD	**0.885**	**0.787-0.996**	**0.043**
	Age	0.993	0.945-1.044	0.789
	BMI	1.075	0.966-1.196	0.188
	Hypertension	0.602	0.300-1.209	0.154
	DM	0.816	0.355-1.878	0.633
	PSA	**1.009**	**1.001-1.016**	**0.023**
	ISUP	**1.527**	**1.155-2.019**	**0.003**
	T stage	**1.581**	**1.097-2.277**	**0.014**
	M	0.940	0.264-3.341	0.924
**4**	**Model 3+NHT**			
	RDW-SD	**0.870**	**0.770-0.982**	**0.024**
	Q1	reference		
	Q2	0.920	0.371-2.285	0.858
	Q3	0.634	0.260-1.548	0.317
	Q4	**0.362**	**0.133-0.989**	**0.048**
	Age	0.995	0.946-1.046	0.840
	BMI	1.089	0.976-1.216	0.128
	Hypertention	0.599	0.296-1.213	0.155
	DM	0.882	0.376-2.068	0.773
	PSA	**1.013**	**1.004-1.022**	**0.005**
	ISUP	**1.589**	**1.197-2.110**	**0.001**
	T stage	**1.655**	**1.138-2.406**	**0.008**
	M	0.883	0.244-3.198	0.850
	NHT	**0.410**	**0.187-0.896**	**0.025**

Multivariate regression stepwise models are shown. The bold value indicated statistical significance. The dependent variable was LVI of PCa. Model 1 was unadjusted. Model 2 corrected for covariates including age, BMI, hypertension, and diabetes mellitus. Model 3 additionally corrected for PSA, ISUP, stage T, and M based on Model 2; Model 4 additionally corrected for NHT based on Model 3. RDW, red blood cell distribution width; LVI, lymphovascular invasion; RDW-SD, red blood cell distribution width-standard deviation; BMI, body mass index; DM, diabetes mellitus; PSA, prostate-specific antigen; ISUP, International Society of Urological Pathology; M, metastasis; NHT, neoadjuvant hormonal therapy.

## Discussion

Our current study firstly investigated the correlation between clinicopathological parameters and LVI in PCa patients who underwent RP, and demonstrated that PSA, stage, and grade were independent risk factors for LVI,

while RDW was independently and negatively associated with the presence of LVI among all whole blood parameters. The results have clinical implications for the prediction of PCa aggressiveness using routine blood tests.

There are multiple lines of evidence supporting that LVI is an independent predictor of BCR and progression and has been associated with metastasis and decreased survival after RP (11, 15, 27–31). The pathological diagnosis of LVI maily depends on HE staining and it can be confirmed by endothelial-associated markers, although this is not often necessary (www.cap.org/protocols-and-guidelines/cancer-reporting-tools/cancer-protocoltemplates). It is reported that LVI exists in 5%-41.7% of RP specimens (32). The wide range of LVI can be explained by the evident difference of severity distribution in different PCa subjects and/or different histological criteria by which LVI is defined. In our study, LVI was presented in 15.2% of the RP specimens, which was within the percentage range of the mentioned data. Our data showed that PCa patients with LVI had higher risk including higher PSA levels, more advanced stage and grade, compared to their counterparts without LVI. These findings were robust and reliabe, in line with the previous studies (15, 32), strongly indicating that LVI is an adverse clinicopathological characteristic. The regression analysis further confirmed that the three parameters were independent risk factors for LVI. It should be noted that percentage of lymph node invasion (LNI) in LVI cases was significantly higher than that in non-LVI cases (22.6% vs. 4.1%), suggesting that there exists a strong link between LVI and LNI, and LVI maybe the first step in developing to LNI. Moreover, 50 cases out of 53 LVI cases were PNI positive (94.3%) in our study, which was similar to one previous research (103/106 = 97.2%) (13). Recently, the role LVI and PNI double positive in prognosis attracted attention in some maliganicies (33, 34), and it need further research in PCa.

Systemic inflammation has been recognized as a part of tumor immune TME and played an important role in the invasion and migration of many solid tumors (35). Changes in peripheral WBC counts and derived inflammatory indicators can reflect inflammatory responses in cancer patients to some extent (35, 36). However, in our study, we indeed did not find significant difference in any kind of WBC or inflammatory parameters including N/L, P/L, SII, L/M, or SIRI. The negative result is likely due to an immunologically cold TME with minimal immune cell infiltration, which is the characteristics of PCa (37).

New urinary and serum biomarkers have been explored in recent years to overcome the current limitations of early detection and outcome prediction of PCa (38, 39). In our study, among all whole blood parameters, only RDW-SD in the LVI group was significantly higher than that in non-LVI group. RBCs are the most common type of blood cells, RDW is based on the width of the RBC volume distribution curve and indicates the size variation of RBCs with larger values indicating greater variability. RDW is usually used for differential diagnosis of anemias in laboratory hematology (40). For example, RDW is elevated when there is ineffective RBC production or increased red cell destruction, such as bone marrow depression and nutritional deficiency (e.g., iron, vitamin B12, or folic acid) (41). RDW was also been found to correlate with inflammation status (40, 41). Recently, growing evidence indicated that RDW has the potential to be a prognostic factor in a variety of cancers (21). There were few studies regarding the relationship between RDW and PCa prognosis or diagnosis. Recently, Cheng J et al. (42) reported that PCa patients with higher pre-treatment and post-treatment RDW levels had poorer 3-year overall survival and cancer-specific survival. One study reported that RDW could be used to develop a novel nomogram to predict the risk of positive biopsy for patients with gray area of PSA (43). In addition, Fukuokaya W, et al. (44) reported that high RDW is an independent predictor of worse treatment outcomes in patients with CRPC treated with androgen receptor axis-targeted agents. However, in our study, RDW-SD in the LVI group was significantly higher than that in non-LVI group, suggsting that higher RDW-SD maybe an inhibitory factor for the occurrence of LVI. Furthermore, the multivariate logistic regression analysis validated that RDW was significantly and negatively correlated with the presence of LVI. We speculated that the high heterogeneity may induce some large size RBCs can’t get into the small vessels and thereafter reduce the adhesion and invasion of PCa cells. Our data may provide new clues for the mechanism of LVI and our hypothesis needs further exporation. Moreover, our result showed that NHT was the other parameter that was negatively and independently correlated with the presence of LVI, indicating that NHT could lessen the aggressiveness of PCa cells in lymphatic or vascular vessels. There were three main limitations in our study. First, RDW may be influenced by various and different pre-existing factors including patient’s specific comorbidities and lifestyle. We have strict exclusion criteria in our study to prevent selection bias as much as possible, however, it is hard to eliminate all bias. For example, it is reported that male testosterone levels have impact on RBC metabolism, variation and immune status (45–47), which may directly or indirectly infuence the RDW level, complete data of testosterone levels in PCa patients in our future collection or in other centers could be added to our multivariate logistic regression analysis. Secondly, the data was analyzed at a single center, and the results needed validation from different institutions in a large sample size. Thirdly, the exact role of RDW on LVI was not explored in the current analysis.

## Conclusions

In conclusion, this study first demonstrated the clinicopathological characteristics including whole blood parameters and derived inflammatory indicators between LVI and non-LVI of PCa patients who underwent RP, and finally found that RDW was negatively and independently associated with the presence of LVI. Our study may provide important clues to reveal the underlying mechanism of LVI in PCa.

## Data availability statement

The raw data supporting the conclusions of this article will be made available by the authors, without undue reservation.

## Ethics statement

The studies involving human participants were reviewed and approved by Ethical Committee of National Cancer Center. Written informed consent for participation was not required for this study in accordance with the national legislation and the institutional requirements.

## Author contributions

Conceptualization: FW, NX. Formal analysis: FW, FL, FY, SH. Investigation: FW, JL, FL. Methodology: FW, JL, FL. Pathology analysis: JL. Supervision: NX. Writing - original draft: FW. Writing - revision & editing: FW, JL, FL, NX. All authors read and approved the final manuscript. All authors contributed to the article and approved the submitted version.

## Funding

This work was supported by grants from National Key R&D Program of China (Grant No.2022YFE0200800), National Natural Science Foundation of China (Grant No. 81972400), The Capital Health Research and Development of Special Funding (Grant No. 2022-1-4021), the CAMS Initiative for Innovative Medicine (Grant No. 2021-I2M-1-015), and Beijing Hope Run Special Fund of Cancer Foundation of China (no. LC2019B02)

## Conflict of interest

The authors declare that the research was conducted in the absence of any commercial or financial relationships that could be construed as a potential conflict of interest.

## Publisher’s note

All claims expressed in this article are solely those of the authors and do not necessarily represent those of their affiliated organizations, or those of the publisher, the editors and the reviewers. Any product that may be evaluated in this article, or claim that may be made by its manufacturer, is not guaranteed or endorsed by the publisher.

## Abbreviations

LVI, lymphovascular invasion; RP, radical prostatectomy; PCa, prostate cancer;BMI, body mass index; NHT, neoadjuvant hormonal therapy; pT, pathological stage T; pN, lymph node status; ISUP, international society of urological pathology; PSM, positive surgical margin; PSA, prostate-specific antigen; RDW, red cell distribution width; PNI, perineural invasion; BCR, biochemical recurrence; TME, tumor microenvironment; RBC, red blood cell; WBC, white blood cell; PLT, platelet; N/L, neutrophil-to-lymphocyte ratio; P/L, platelet-to-lymphocyte ratio; SII, systemic immune-inflammation index; L/M, lymphocyte-to-monocyte ratio; SIRI, systemic inflammation response index; Hb, hemoglobin; Hct, hematocrit; MCV, mean corpuscular volume; MCH, mean corpuscular hemoglobin; MCHC, mean corpuscular hemoglobin concentration; SD, standard deviation; MPV, mean platelet volume; P-LCR, platelet-large cell ratio; PDW, platelet distribution width; HE, hematoxylin-eosin; LNI, lymph node invasion.
